# Evaluating and predicting the effectiveness of farmland consolidation on improving agricultural productivity in China

**DOI:** 10.1371/journal.pone.0198171

**Published:** 2018-06-06

**Authors:** Yeting Fan, Xiaobin Jin, Xiaomin Xiang, Le Gan, Xuhong Yang, Zhihong Zhang, Yinkang Zhou

**Affiliations:** 1 School of Geographic and Oceanographic Sciences, Nanjing University, Nanjing, Jiangsu, China; 2 Key Laboratory of Coastal Zone Exploitation and Protection, Ministry of Land and Resources, Nanjing, Jiangsu, China; 3 Natural Resources Research Center, Nanjing University, Nanjing, Jiangsu, China; 4 China Land Surveying and Planning Institute, Ministry of land and resources, Beijing, China; University of Bonn, GERMANY

## Abstract

Food security has always been a focus issue in China. Farmland consolidation (FC) was regarded as a critical way to increase the quantity and improve the quality of farmland to ensure food security by Chinese government. FC projects have been nationwide launched, however few studies focused on evaluating the effectiveness of FC at a national scale. As such, an efficient way to evaluate the effectiveness of FC on improving agricultural productivity in China will be needed and it is critical for future national land consolidation planning. In this study, we selected 7505 FC projects completed between 2006 and 2013 with good quality Normalized Difference Vegetation Index (NDVI) as samples to evaluate the effectiveness of FC. We used time-series Moderate Resolution Imaging Spectroradiometer NDVI from 2001 to 2013, to extract four indicators to characterize agricultural productivity change of 4442 FC projects completed between 2006 and 2010, i.e., productivity level (PL), productivity variation (PV), productivity potential (PP), and multi-cropping index (MI). On this basis, we further predicted the same four characteristics for 3063 FC projects completed between 2011 and 2013, respectively, using Support Vector Machines (SVM). We found FC showed an overall effective status on improving agricultural productivity between 2006 and 2013 in China, especially on upgrading PL and improving PP. The positive effect was more prominent in the southeast and eastern China. It is noteworthy that 27.30% of all the 7505 projects were still ineffective on upgrading PL, the elementary improvement of agricultural productivity. Finally, we proposed that location-specific factors should be taken into consideration for launching FC projects and diverse financial sources are also needed for supporting FC. The results provide a reference for government to arrange FC projects reasonably and to formulate land consolidation planning in a proper way that better improve the effectiveness of FC.

## Introduction

Food production in China has increased markedly over past quarter-century [1]. With China entering a new era of rapid urbanization, the issues such as population growth, climate change, biodiversity loss and resource depletion have posed a great threat to food security [2–5]. The primary approach used to guarantee steady and sustainable food production is to convert more land into farmland and to improve the quality of existing farmland [2]. However, farmland in China has decreased by 8.22 million ha during 1997 to 2013 [6], and land degradation has accounted for more than 40% of the total area lost [7]. In this situation, modern land consolidation in China was initiated from 1997.

Land consolidation in China is closely related to the policy of Farmland Requisition-Compensation Balance, which focuses on reclaiming the same quantity and quality of new farmland to offset losses in farmland area [8–10]. More than 5.45 million ha farmland has been supplemented by land consolidation since 1997. As a type of land consolidation, the main target of farmland consolidation (FC) is improving agricultural productivity and increasing food production through optimizing the structure and layout of land use, improving infrastructure in support of agriculture and ameliorating soil quality, etc. In 2006, 116 national basic farmland (e.g., high-quality farmland which is prohibited conversion to non-agricultural activities, see [10–12]) protection demonstration zones were designated by the Chinese government, which indicates basic farmland construction has become an important content of land consolidation, and improving the quality of farmland became the core of land consolidation [13]. From 2011 to 2015, China has developed 26.87 million ha high-standard basic farmland (e.g., contiguous high-quality farmland with supporting facilities as well as high and stable productivity, see [12]) and added 37.4 billion kg food production capacity [14]. From the perspective of statistical data analysis, the effectiveness of FC on improving agricultural productivity is remarkable [13, 14].

Previous studies on effectiveness evaluation of FC mainly focused on yield monitoring [15], potential productivity evaluation [8, 16, 17], projects impact assessment [18, 19], policy assessment [20, 21], farmland fragmentation [22, 23]. These studies have presented FC can make a positive effect on improving agricultural productivity. Wu et al. (2005) evaluated the effectiveness of land consolidation projects from 227 Chinese farm households and indicated land consolidation has contributed 1.52% to household crop output [18]. Pašakarnis and Maliene (2010) showed land consolidation could improve working conditions in agriculture and improve agricultural production in most European countries [21]. Abdollahzadeh et al. (2012) found FC is regarded as a suitable pathway in Iran to reduce land fragmentation so that decrease production input costs (labor, fuel, and machinery) and improve agricultural productivity [22]. Song et al. (2014) revealed the average Natural Quality Grades of cultivated land gained from land consolidation during 1999 to 2008 was 8.39 and have a prominent ability to improve the potential land productivity [8]. However, most of these studies on the effectiveness of FC were conducted at local scale or at project scale, and the results relied heavily on a range of rough data (e.g., statistical data, report summaries or field surveys), which may be of human error and themselves be not reliable [24, 25]. As we know, FC in China has already expanded to nationwide scale, it is necessary to conduct a comprehensive and systematic evaluation on the effectiveness of FC in improving agricultural productivity at national scale using finer data.

Compared with traditional research methods, remote sensing data provide the most effective method for identifying the change of agricultural productivity influenced by FC across broad geographic extents [26, 27]. An approach using time-series Normalized Difference Vegetation Index (NDVI), which has been derived from the satellite based Moderate Resolution Imaging Spectroradiometer (MODIS), may augment research capabilities based on its high resolution and continuous detection [28]. Some studies have showed a positive relationship exists between NDVI and crop yield (e.g., wheat, soybean, see [29–31]). MODIS NDVI time series data have also been used to demarcate major crop types and characterize cropping systems using in a special area [32, 33]. As a result, NDVI time-series data have been proven to be a useful predictor for estimating agricultural productivity change, and have successfully applied in agriculture monitoring [26, 29, 34], yield estimation [30, 31, 35], and land cover change detection [32, 33]. For FC projects with parcel boundary spatially, it is a feasible way to identify and characterize the change of agricultural productivity influenced by FC using NDVI time series data. However, to our knowledge, few studies have extracted distinct indicators to characterize the change of agricultural productivity using remote sensing data and have evaluated the effectiveness of FC on improving agricultural productivity at a nationwide scale [36–39].

Jin et al. (2017) have estimated the average annual change rate of agricultural productivity for land consolidation projects completed between 2006 and 2010 in China based on NDVI time series data [37]. Du et al. (2018) have assessed the effectiveness of land consolidation projects completed in 2006 and 2007 for improving and stabling production in China based on NDVI time series data [39]. However, their studies only focused on the change of productivity mean level influenced by land consolidation, which is lack of consideration on different forms of agricultural productivity change, including productivity potential and production intensity. Land consolidation projects launched only in the early period were considered in the above two studies (e.g., 2006 and 2007) and were used to evaluate the effectiveness of land consolidation. How to predict the effectiveness of intended projects in the future land consolidation planning, the studies haven’t answer the question, which was of great importance for government to make decision for land consolidation projects arrangement in the future. In this study, a total of 7505 FC projects with parcel boundary information completed between 2006 and 2013 were selected, among which 4442 projects completed between 2006 and 2010 (hereinafter, former projects) as well as 3063 projects completed between 2011 and 2013 (hereinafter, later projects). We extracted four indicators to characterize the change of agricultural productivity, i.e., productivity level (PL), productivity variation (PV), productivity potential (PP), and multi-cropping index (MI) using NDVI time series data. As the time needed for soil to recover and become stable since the disturbance caused by the engineering construction of projects, the recovery and improvement of agricultural productivity has a time lag (usually 3 years) following FC [40, 41]. It is difficult to accurately evaluate the effectiveness of later projects on improving agricultural productivity using NDVI time series data. As such, we introduced a machine learning algorithm, i.e., Support Vector Machines (SVM) to predict the effectiveness of later projects on improving agricultural productivity based on a combination of the effectiveness of former projects and potential factors that could influence FC.

The main objectives of our study were as followings: (1) identify and evaluate the effectiveness of former projects on improving agricultural productivity using NDVI time series data, (2) predict the effectiveness of later projects on improving agricultural productivity using SVM, and (3) conducted a comprehensive evaluation on the effectiveness of FC on improving agricultural productivity. In the end, we also discussed the probably reasons for non-effective projects and provided some implications for improving the effectiveness of FC.

## Data and methods

### Data sources and pre-processing

#### Farmland consolidation projects information

**Fig 1:**
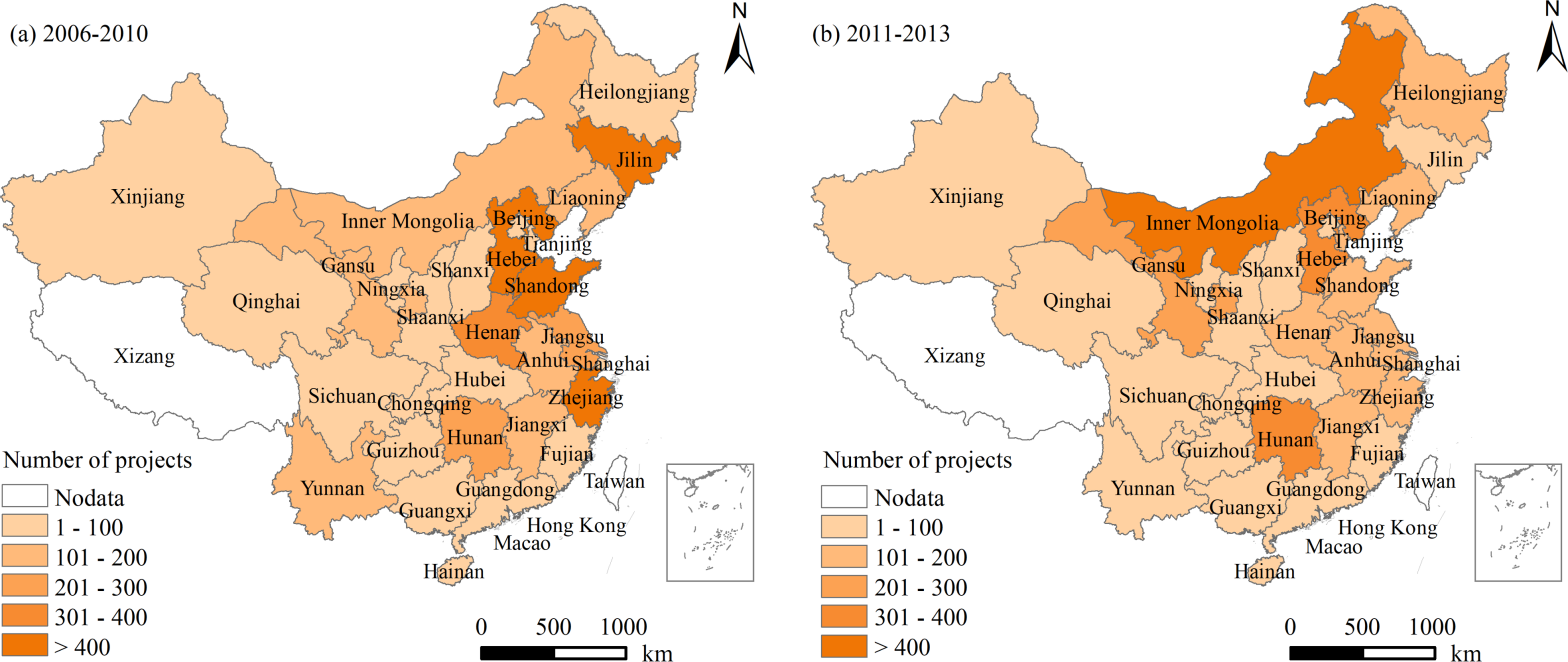
Spatial distribution of the number of farmland consolidation projects completed between 2006 and 2013.

FC projects were obtained from the “National Rural Land Consolidation Monitoring and Regulation System” maintained by the Ministry of Land and Resources of China. All the 7505 completed FC projects selected for analysis all had clear spatial boundaries. Detailed information of FC projects included project location, parcel boundary, parcel area and completion acceptance date. For further analysis, the former projects and the later projects were separated and summarized as shown in Fig 1. The locations of the projects were mapped in S1 Fig.

#### NDVI time series data

NDVI time series data employed in this study were MOD13Q1 Terra vegetation index data obtained from the NASA Reverb website (http://reverb.echo.nasa.gov/). The data is a 16-day maximum value composite with 250m spatial resolution from January 1, 2001 to December 31, 2013. For each compositing window (e.g., 2001001), 28 NDVI image scenes for the study area were mosaiced and re-projected to Albers Equal Area Conic projection. For the period of 2001 to 2013, a total of 8372 NDVI scenes were processed to provide time series data for the former projects in the study area.

#### Influencing factors for agricultural productivity change

Based on existing literatures [17, 37, 42, 43, 44] and indicator selection criteria of comprehensiveness, independence, diversity, and feasibility, we proposed sixteen explanatory variables as potential influencing factors related to agricultural productivity change. They respectively were: (1) natural factors, including elevation, slope, average annual precipitation, the rate of crop disaster area; (2) socioeconomic factors, including per capita GDP, urbanization rate, population density, and road density; (3) land resource factors, including per capita area of farmland, farmland quality, multi-cropping index, and area of farmland reserve resources; (4) project property factors, including plot size, shape index, project investment intensity, and the nearest distance to the county center. Detailed information of these factors, including source, resolution and unit are provided in Table 1.

**Table 1:** Description of potential factors influencing changes in agricultural productivity during the period of farmland consolidation.

Types	Variables	Source	Resolution	Unit
Natural factors	Elevation	USGS DEM product	1km × 1km	m
	Slope		1km × 1km	°
	Average annual precipitation	China Meteorological Data Sharing Service System	500m × 500m	mm
	The rate of crop disaster area	Statistics Yearbook of China	City-level	%
Socioeconomic factors	Per capita GDP	Statistics Yearbook of China	County-level	USD/person
	Urbanization rate	The Sixth National Census in China	County-level	%
	Population density		County-level	Person/km^2^
	Road density	Obtained from Baidu map	1 km × 1 km	km/km^2^
Land resource factors	Per capita area of farmland	Nation Earth System Science Data Sharing Infrastructure of China	County-level	ha
	Farmland quality	National Agricultural Land Grading Results in China	1:500k	grade
	Multi-cropping index	Comprehensive Agricultural Regionalization of China	1:4000k	%
	Area of farmland reserve resources	National Farmland Reserve Resources Survey in China	City-level	ha
Project property factors	Plot size	Obtained from attribute table of projects	Project-level	ha
	Shape index		Project-level	—
	Investment intensity		Project-level	USD/ha
	Nearest distance to the county center	Obtained from the distance between project parcel center and county center	Project-level	km

### Methods

**Fig 2:**
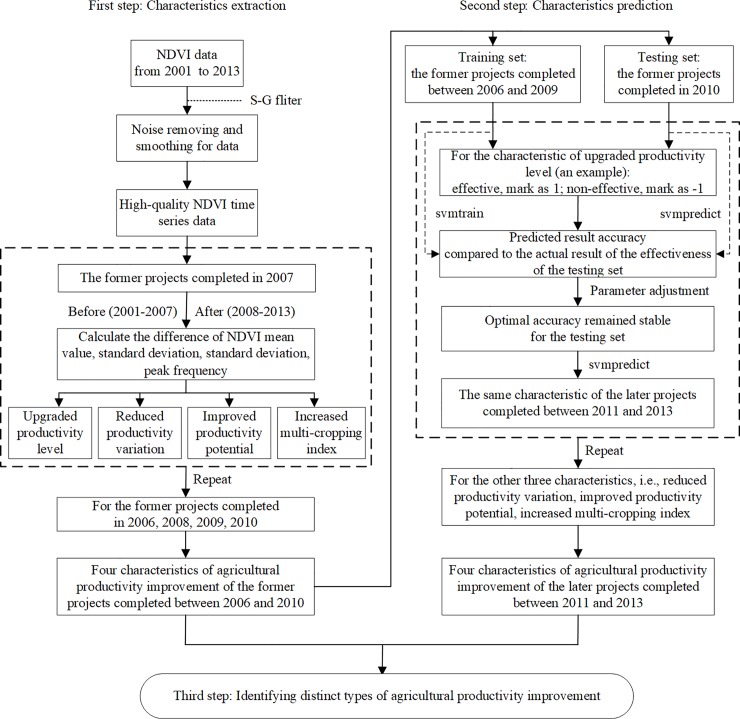
An overall framework of analysis method in the study.

Three analysis steps were conducted in this study. First is the characteristics extraction of agricultural productivity improvement for the former projects using NDVI time series data, second is the prediction of agricultural productivity improvement for the later projects using SVM, the third is distinct types identification of agricultural productivity improvement. The overall framework of analysis methods is shown in Fig 2.

#### Extracting the indicators characterized agricultural productivity improvement of the former projects

**Fig 3:**
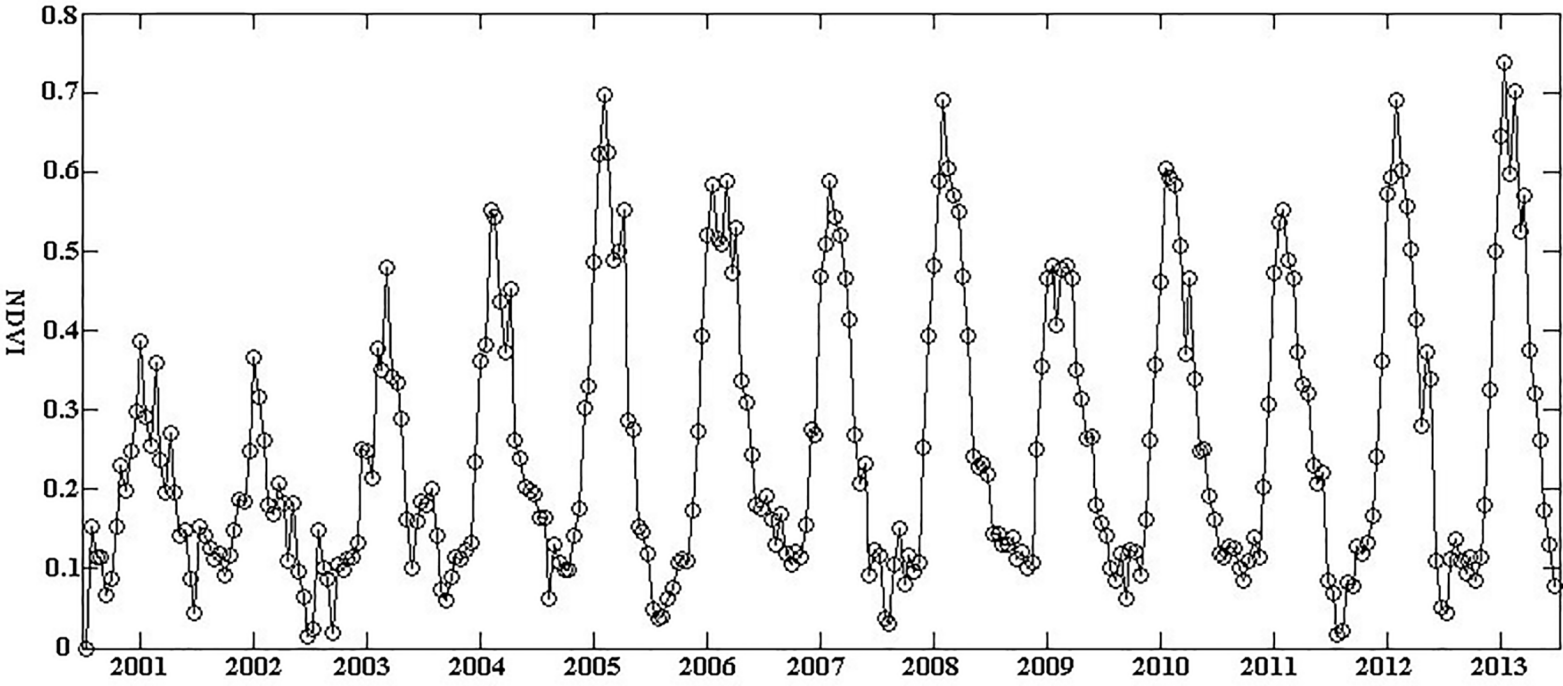
NDVI time series data of a typical farmland consolidation project derived from 2001 to 2013.

The spatial boundaries of FC projects were used to extract NDVI values. NDVI data were obtained with an interval of 16-day from the satellite and include 23 composite scenes in each year. Thus, we extracted 23 NDVI values to build an annual NDVI time series. To ensure the reliability of the data used for analysis, we set data screening criteria to meet both of the following requirements simultaneously, i.e., quality assessment of data is reliable (i.e., Reliability Index (RI) ≤1) and no more than five missing NDVI values for each parcel. As such, the parcel area of each project selected in this study was larger than 100 ha since small parcels (i.e., <100ha) covered limited number of NDVI pixels. We also used an S-G filter of TIMESAT3.2 [45] to exclude poor-quality (RI>1) NDVI pixel covered with clouds, water, snow, and ice. For each project parcel, we averaged NDVI values at a 16-day composite image and then repeated this procedure 23 times to obtain NDVI composite values for each year. NDVI time series data of a typical project was shown in Fig 3.

FC is the most comprehensive farmland adjustment activity, addressing a range of land engineering measures which are aimed at optimizing farmland layout, improving irrigation and water conservation infrastructure as well as soil fertility. Agricultural productivity change, manifested as differences in crop yield [17], the objectives of FC on improving agricultural productivity usually include increased average yield, reduced yield fluctuation, improved maximum yield, increased efficiency in agricultural production, which are related to productivity level, productivity variation, productivity potential and harvest time. Jin et al. (2017) and Du et al. (2018) have also showed explicit methods to characterize the agricultural productivity (e.g., mean annual productivity, productivity variation) using NDVI time series data [37, 39]. Estel et al. (2016) have extracted four indicators to characterize cropping systems in Europe, i.e., cropping frequency, multi-cropping, fallow cycles, and crop duration ratio, based on MODIS NDVI time series data, which highlighted the importance and usefulness for satellite image time series to characterize spatial patterns in agricultural productivity [26]. Ding et al. (2015) have employed difference algorithm to extract the multi-cropping index of cropland based on NDVI data from1999 to 2013 [46].

Based on the previous studies and the nature of agricultural productivity change, in this study, we calculated four indicators using NDVI time series data to characterize different forms of agricultural productivity change. The indicators are the difference of NDVI mean value, NDVI standard deviation, NDVI maximum value and NDVI peak frequency before and after the completion year of the project, respectively, to indicate the change of productivity level (PL), productivity variation (PV), productivity potential (PP) and multi-cropping index (MI), respectively. For example, for a project completed in 2007, the four indicators were calculated as following formulas.

$$MEN_{change}={\bar{NDVI}}_{after}‑{\bar{NDVI}}_{before}$$ (1)

where ${\bar{NDVI}}_{after}$, ${\bar{NDVI}}_{before}$ represent NDVI mean value from 2008 to 2013 and from 2001 to 2007, respectively; MEN_*change*_ represents the difference value of NDVI mean value before and after FC.

$$SDN_{change}=\sigma (NDVI_{after})‑\sigma (NDVI_{before})$$ (2)

where σ(NDVI_*after*_), σ(NDVI_*before*_) represent NDVI standard deviation from 2008 to 2013 and from 2001 to 2007, respectively; SDN_*change*_ represents the difference value of NDVI standard deviation before and after FC.

$$MAN_{change}=\max(NDVI_{after})‑\max(NDVI_{before})$$ (3)

where max(NDVI_*after*_), max(NDVI_*before*_) represent NDVI maximum value from 2008 to 2013 and from 2001 to 2007, respectively; MAN_*change*_ represents the difference value of NDVI maximum value before and after FC.

$$PFN_{change}=PF(NDVI_{after})‑PF(NDVI_{before})$$ (4)

where PF(NDVI_*after*_), PF(NDVI_*before*_) represent NDVI peak frequency from 2008 to 2013 and from 2001 to 2007, respectively; PFN_*change*_ represents the difference value of NDVI peak frequency before and after FC.

**Fig 4:**
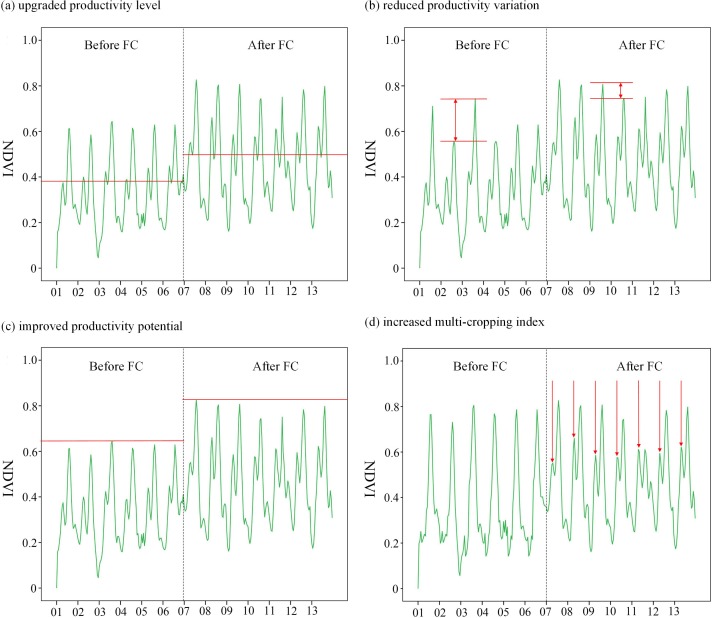
Four indicators used to characterize agricultural productivity change using NDVI time series data. FC represents farmland consolidation. 01–13 in the horizontal axis represent the years of 2001–2013, respectively.

Among above four formulas, $\bar{NDVI}$, σ(NDVI), max(NDVI) could be calculated by simple mathematical calculation; PF(NDVI) was calculated following the existing literatures [46, 47]. The criteria used to identify the effectiveness of FC on the four characteristics of agricultural productivity improvement are as followings. MEN_*change*_ > 0, it indicated the project was effective on updating PL, otherwise was non-effective; SDN_*change*_ < 0, it indicated the project was effective on reducing PV, otherwise was non-effective; MAN_*change*_ > 0, it indicated the project was effective on improving PP, otherwise was non-effective; PEN_*change*_ > 0, it indicated the project was effective on increasing MI, otherwise was non-effective. Each characteristic scenarios of agricultural productivity improvement extracted by NDVI time series data was visualized and mapped in Fig 4.

#### Predicting the characteristics of agricultural productivity improvement of the later projects

Support vector machines (SVM) was first proposed by Cortes and Vapnik based on statistical theory in 1995 [48]. SVM provides a supervised learning method based on structural risk minimization which minimizes expected error of a learning model that identifies hyperplanes (or decision planes) based on training data for classification and prediction problems [49, 50]. The decision boundaries measure similarities between objects (kernels), i.e., data features are transformed into multidimensional space where the features can be separated into optimal different predictions or classes more easily by the hyperplanes [51]. To date, SVM has been successfully applied in many classification and prediction studies, such as land cover classification [52, 53], vegetation type recognition [54, 55], and crop yield prediction [56, 57].

In this study, we divided the former projects into training set (2982 projects completed between 2006 and 2009) and testing set (1460 projects completed between 2010). Training set was used to learn for optimal hyperplane, and testing set was used to test the accuracy of the learning result. Before learning, for each characteristic of agricultural productivity change, a project was given a value of positive 1 or negative 1 to indicate the effectiveness of the project on improving agricultural productivity. For example, a project was given a value of positive 1 if it was of upgraded PL, otherwise the project was given a value of negative 1; then we classified all the values into a set *y*_*i*_ = {1, −1}, *i* = 1, 2, …, 4442. The same classification steps for the characteristics of PV, PP, and MI. In addition, the later projects were seen as prediction set with unknown characteristics of agricultural productivity change. We also identified the values of 16 potential influencing factors for both the former and later projects, which were defined as feature vectors ***x***_*i*_∈**R**^16^.

LIBSVM [47] was introduced to Matlab 2015a software (MathWorks, https://cn.mathworks.com/) for SVM implementation. *C*-Support Vector Classification (*C*-SVC) was used to solve two-class classification problem. Radial basis function (RBF) was employed to map ***x***_*i*_ into a higher dimensional space, which has been proved to be of high applicability for classification and prediction [52, 55], the function formula is

$$k(x_{i},x_{j})=\exp(-g{\left\|x_{i},x_{j}\right\|}^{2})$$ (5)

where *k*(***x***_*i*_,***x***_*j*_) is the radial basis function, and *g* is the kernel parameter.

Parameters *C* and *g* are both constants and the key parameters which affect the performance of SVM classifier. *C* indicates the penalty degree for misclassification samples, *g* indicates the radial range. We employed cross validation to test the overall accuracy in the model based on debugging repeatedly parameters *C* and *g* using a grid searching approach [52]. The searching steps were as following:

First, we conducted a coarse-searching in a long distance and large range to determine the optimal intervals of parameters *C* and *g* for each characteristic.

Second, within above optimal intervals of parameters *C* and *g*, we conducted a fine-searching for parameters *C* and *g* to obtain the corresponding classification accuracy under different groups of parameters *C* and *g*.

Once a high and stable accuracy was obtained for each characteristic in the learning process, we stopped the searching step and finished the running of the model.

#### Identifying distinct types of agricultural productivity improvement influenced by farmland consolidation

Based on the identification of the four indicators of agricultural productivity improvement, we determined seven distinct types of agricultural productivity improvement influenced by FC. As the prime objective of FC, upgraded PL is the most elementary and important indicator characterized agricultural productivity improvement [17, 18]. As such, for all 7505 projects completed between 2006 and 2013, we assumed a project was effective in elementary improvement of agricultural productivity if the project was effective in upgrading PL. On this basis, we further determined other six types of agricultural productivity improvement (Table 2).

**Table 2:** Distinct types of agricultural productivity improvement influenced by farmland consolidation.

Type of agricultural productivity improvement	Type abbreviation	Indicator of agricultural productivity improvement			
		Upgraded productivity level	Reduced productivity variation	Improved productivity potential	Increased multi-cropping index
Elementary improvement	EI	Effective	Non-effective	Non-effective	Non-effective
Stability improvement	SI	Effective	Effective	Non-effective	Non-effective
Potential improvement	PI	Effective	Non-effective	Effective	Non-effective
Intensity improvement	II	Effective	Non-effective	Non-effective	Effective
Stability and potential improvement	SPI	Effective	Effective	Effective	Non-effective
Stability and intensity improvement	SII	Effective	Effective	Non-effective	Effective
Optimal improvement	OI	Effective	Effective	Effective	Effective

## Results

### The effectiveness of the former projects on improving agricultural productivity

**Fig 5:**
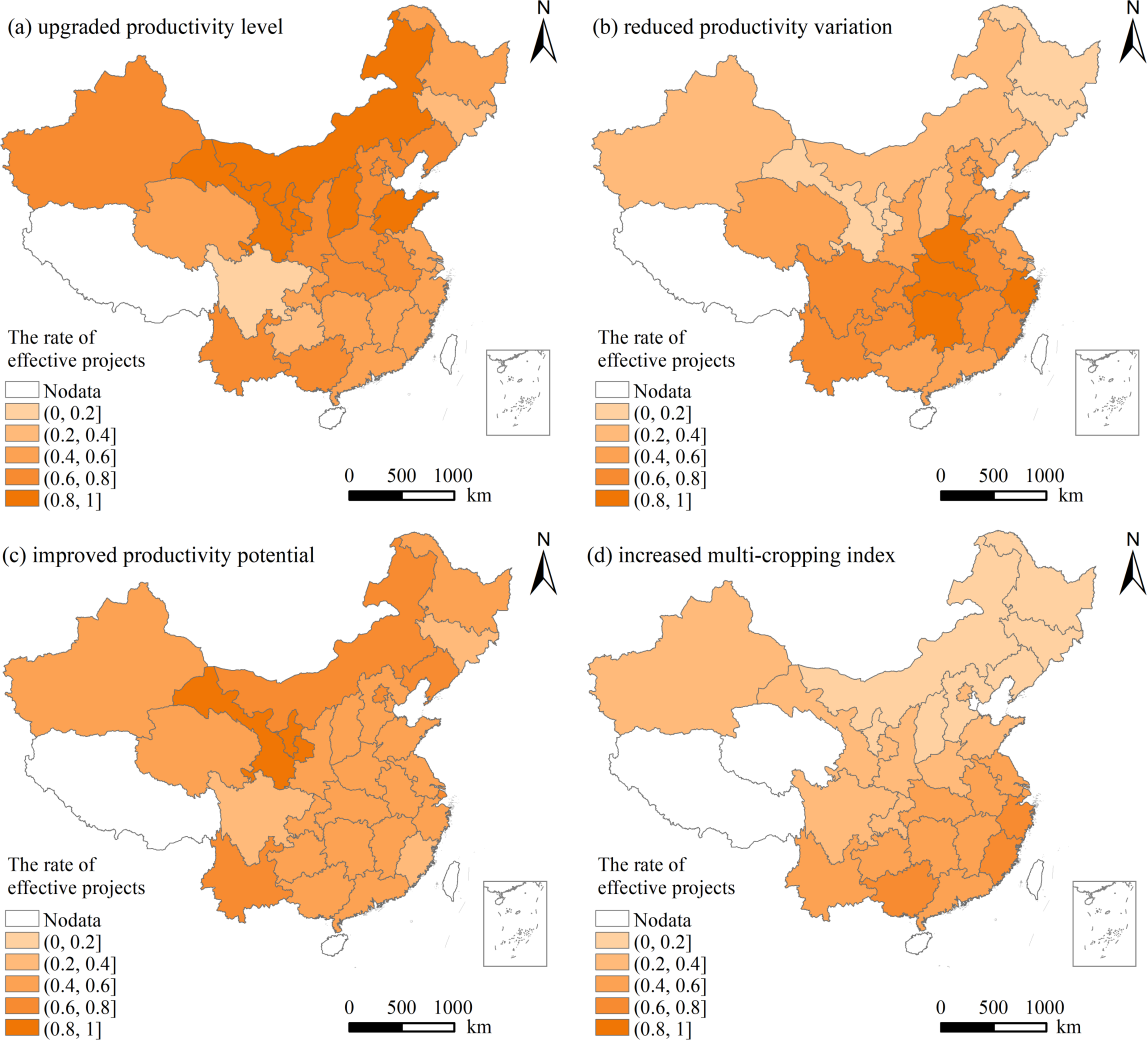
Spatial distribution of the effective projects completed between 2006 and 2010 on improving agricultural productivity in China.

In general, the rates of the former projects with upgraded PL, reduced PV, improved PP, and increased MI were 62.02%, 55.02%, 53.53%, and 33.30%, respectively. The spatial distribution of the effective projects completed between 2006 and 2010 on improving agricultural productivity was shown in Fig 5.

The rates of the projects with upgraded PL and projects with improved PP were both higher in northern China than in southern China. More than half of the provinces in China where the rates of the projects with upgraded PL and improved PP were higher than 50% in the areas. Especially, over 80% of projects in Ningxia and Gansu were effective on both upgrading PL and improving PP. The rates of the projects with upgraded PL in Sichuan, Jilin, Shanghai were less than one third of the total projects in the areas, respectively. Jilin, Ningxia, Heilongjiang, Gansu were the areas where the effectiveness of FC on improving PP was lower than the other areas. The rate of the projects with improved PP in these areas was less than 20%, although nearly 17% of the former projects were located in the areas. The effectiveness of FC on reducing PV was more prominent in the southeast China, specifically, Zhejiang, Hunan, Hubei and Henan. The effectiveness of FC on increasing MI was inferior to that on other three improvements of agricultural productivity. The projects with increased MI was remarkably concentrated in the southeast China, e.g., Zhejiang, Fujian, Guangxi.

### The effectiveness of the later projects on improving agricultural productivity

**Fig 6:**
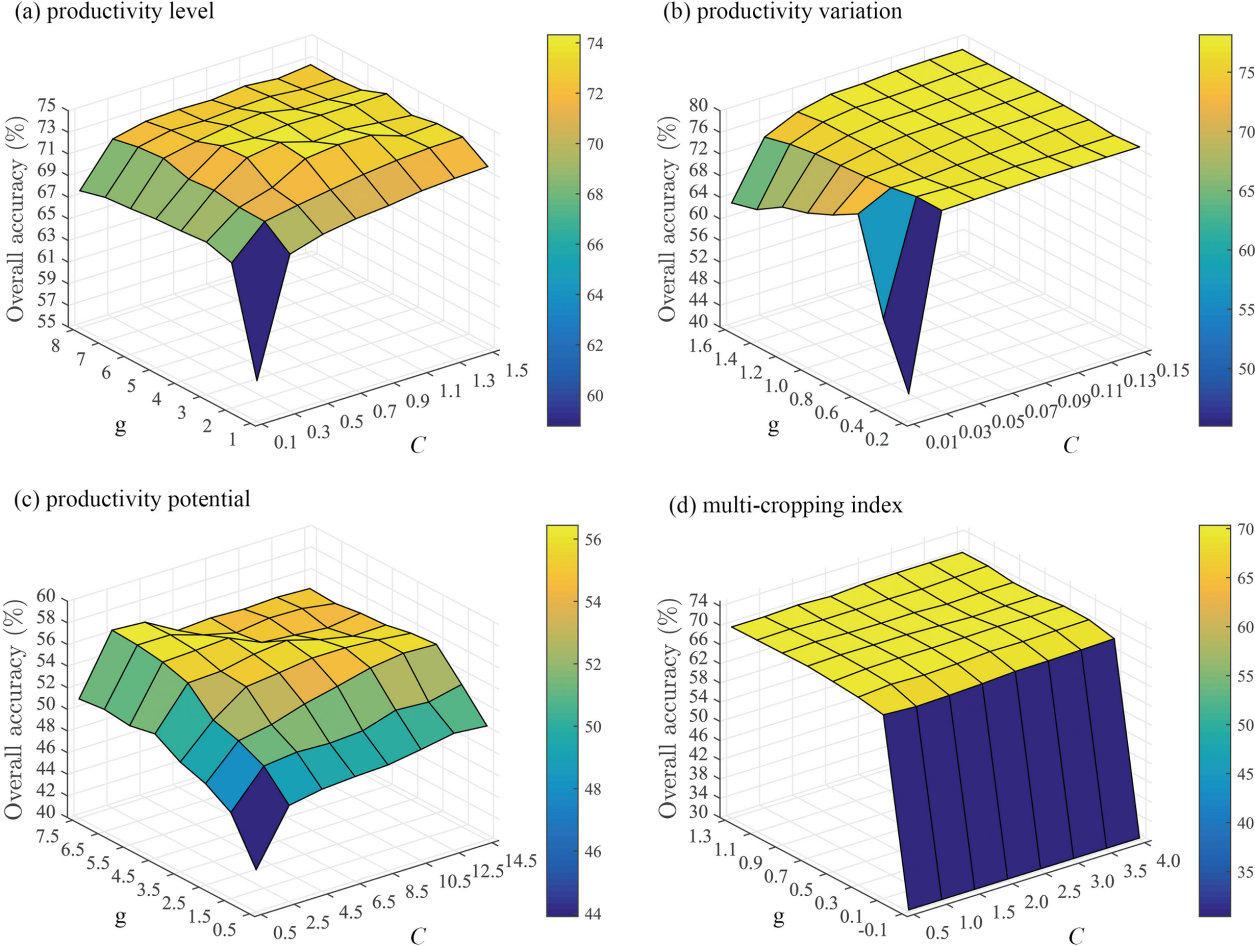
The debugging process of different groups of penalty parameter *C* and kernel parameter *g* using SVM and the consequential overall accuracies.

We identified the unknown effectiveness of the later projects on improving agricultural productivity based on the known effectiveness of the former projects on upgrading PL, reducing PV, improving PP, increasing MI, respectively. The overall accuracies of SVM prediction for the four characteristics based on grid searching were shown in Fig 6.

For each characteristic, we determined a specific range in which overall accuracy remained high and stable under different groups of parameters *C* and *g*. Then we obtained the optimal accuracy in a range of accuracies (Table 3).

**Table 3:** The optimal accuracy predicted by SVM for each characteristic of agricultural productivity change based on the debugging results of parameters *C* and *g*.

Indicator	Parameter *C*	Parameter *g*	Overall accuracy
Change of productivity level	0.9	3.0	74.32%
Change of productivity variation	0.07	1.0	77.33%
Change of productivity potential	9.5	4.5	55.68%
Change of multi-cropping index	2.0	0.7	70.21%

**Fig 7:**
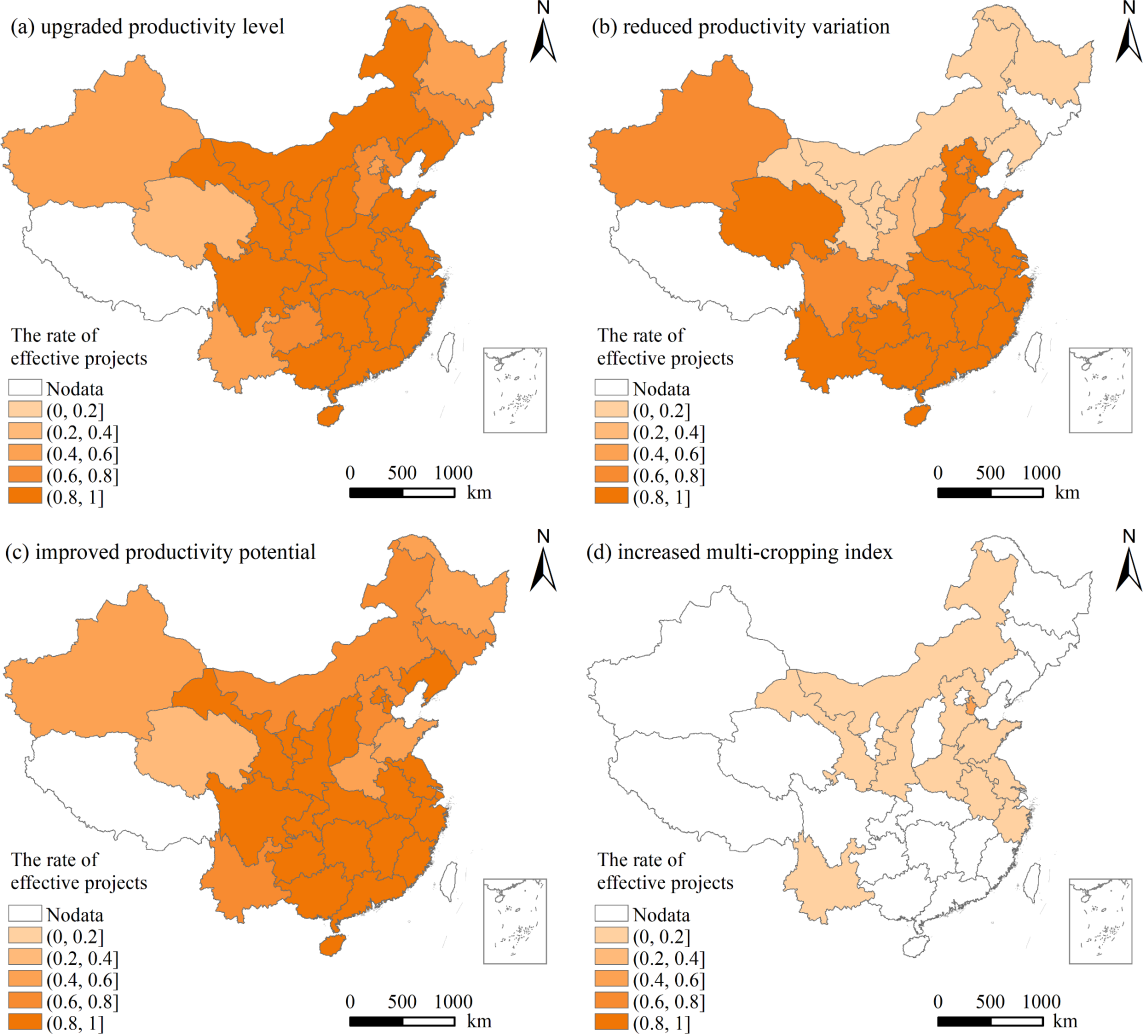
Spatial distribution of the effective projects completed between 2011 and 2013 on improving agricultural productivity in China.

The results showed that the rates of the later projects with upgraded PL, reduced PV, improved in PP, and increased MI were 88.18%, 66.41%, 81.55%, 3.59%, respectively. The effectiveness of the later projects on upgrading PL, reducing PV, improving PP was more prominent than that of the former projects. The spatial distribution of the effective projects completed between 2011 and 2013 on improving agricultural productivity was shown in Fig 7.

The rates of the projects with upgraded PL and projects with improved PP were both more than 20% of the total projects in each province. More than 80% of the total projects in nearly two-thirds of the provinces in China were effective on both upgrading PL and improving PP. Especially, all the projects in Jiangxi, Hunan, Guangxi, Guangdong, Hainan, Shaanxi, Shanghai were effective on upgrading PL, reducing PV and improving PP. The effectiveness of the later projects on reducing PV was more prominent in the southern China and northwest China than in northeast China (e.g., Jilin, Heilongjiang, Inner Mongolia, Liaoning, Gansu, Ningxia). Only 134 projects in the above six provinces were effective on reducing PV, which accounted for less than 14% of the total projects in the areas. The effectiveness of the later projects on increasing MI was lower than that of the former projects. Only 110 projects were effective on increasing MI and were mainly located in less than one third of the provinces in China. The effectiveness of the later projects on increasing MI showed an overall low status. Tianjin was the area where the rate of the projects with increased MI was the highest.

### Comprehensive evaluation on the effectiveness of farmland consolidation on improving agricultural productivity

**Fig 8:**
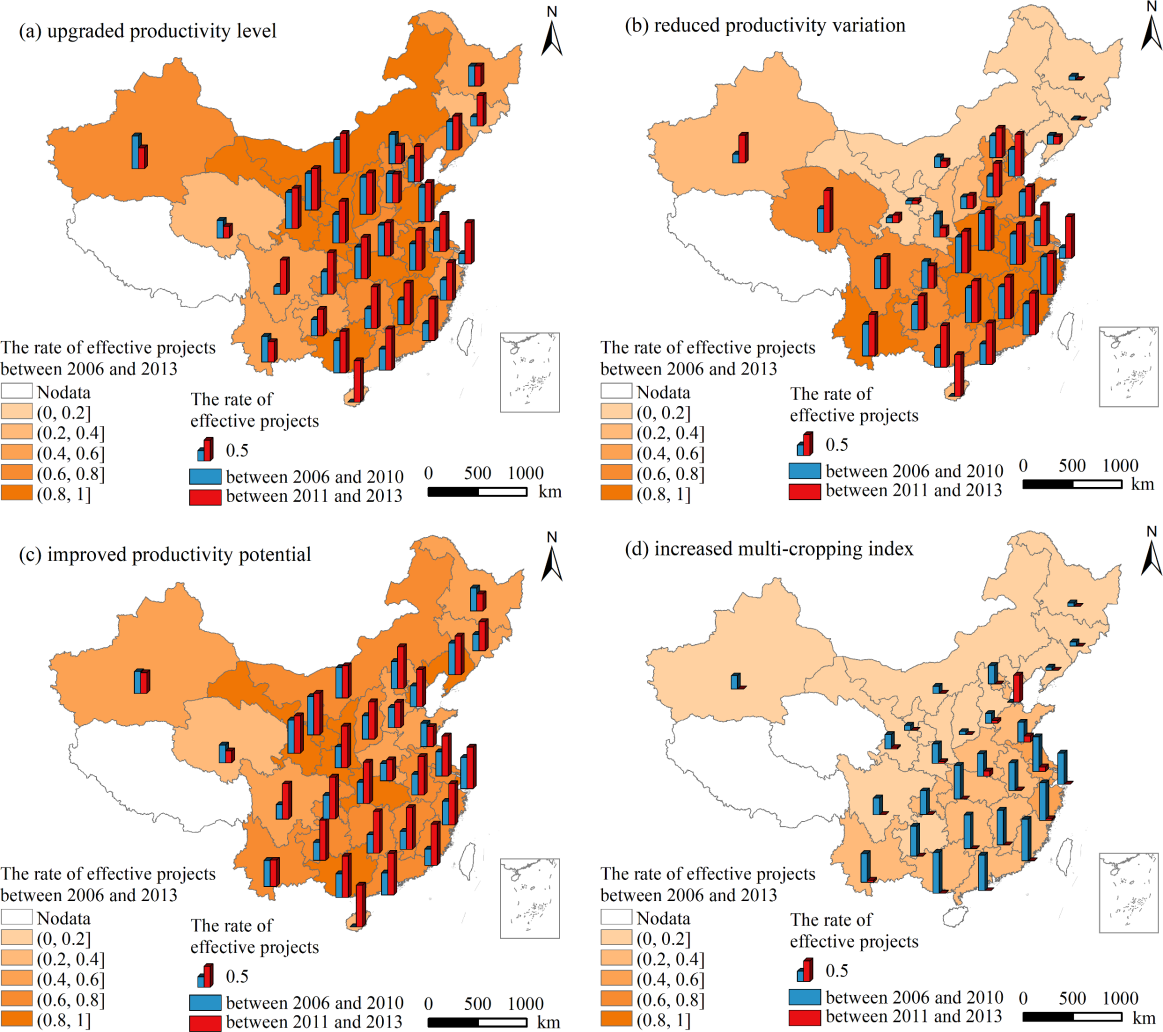
Spatial and temporal distribution of the effective projects completed between 2006 and 2013 in China on improving agricultural productivity in China.

The rates of all 7505 projects with upgraded PL, reduced PV, improved PP, and increased MI were 72.70%, 59.67%, 64.97%, and 21.17%, respectively. The spatial and temporal distribution of the effective projects completed between 2006 and 2013 on improving agricultural productivity was shown in Fig 8.

The spatial and temporal distribution of the projects with upgraded PL was similar to that of the projects with improved PP. The rates of the projects with upgraded PL and the projects with improved PP were both higher in southeast China than in northwest China. The rates of the projects with reduced PV and the projects with increased MI were higher in southern China than in northern China. Compared with the former projects, the rates of the later projects with upgraded PL, reduced PV and improved PP have increased by 26.16%, 11.39%, and 28.02% at a national scale, respectively, whereas the rate of projects with increased MI has decreased by 29.71%. Moreover, the effectiveness of FC on upgrading PL, reducing PV and improving PP in southeast China has become more prominent over time, especially in Hainan, Guangdong, Fujian, Shanghai. However, in Xinjiang and Qinghai, the effectiveness of FC on upgrading PL and improving PP have decreased over time. The effectiveness of FC on increasing MI was getting lower over time in most provinces except for Tianjin.

**Fig 9:**
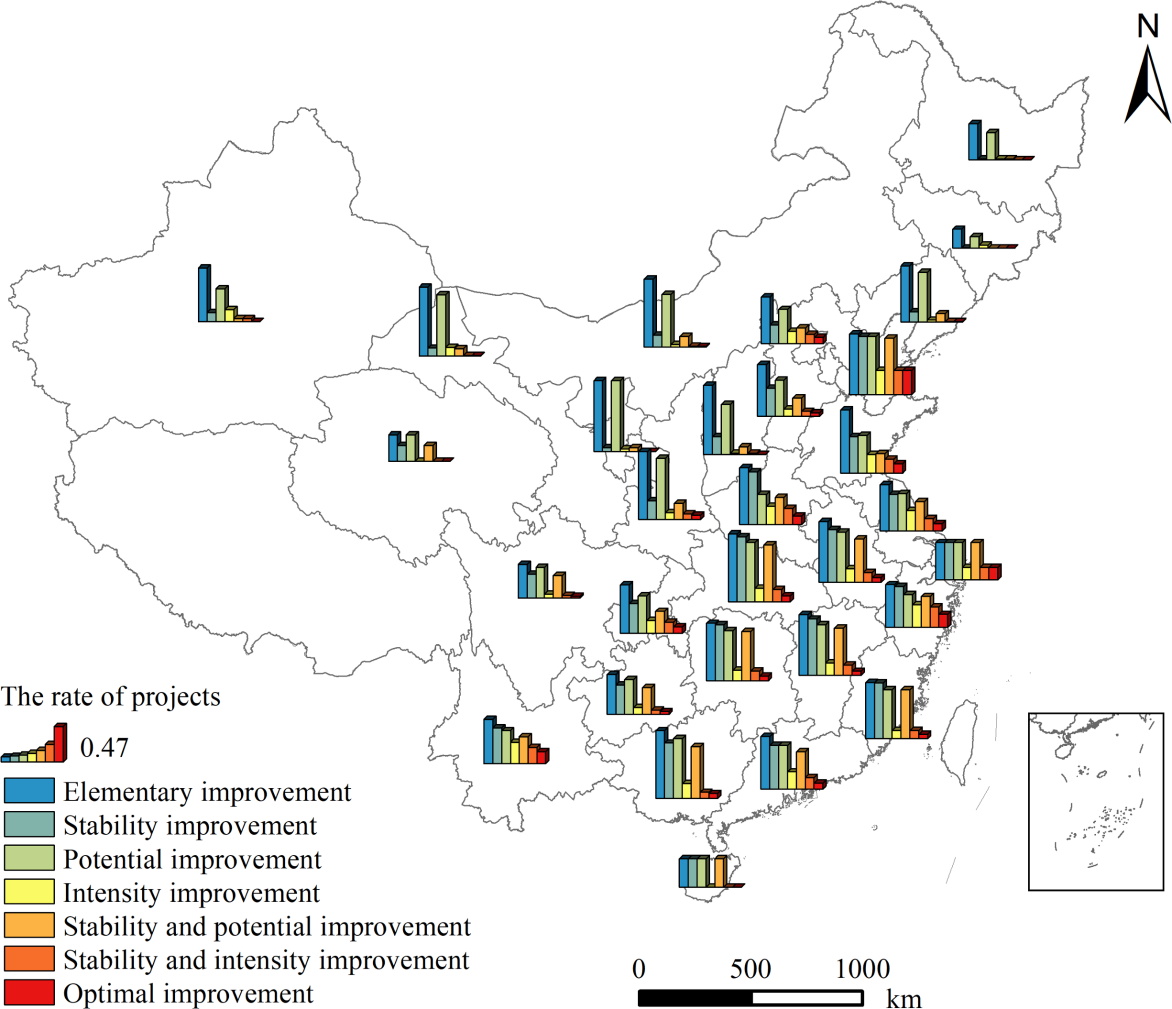
Spatial distribution of the effective projects with distinct types of agricultural productivity improvement completed between 2006 and 2013 in China.

Among all 7505 projects, the rates of the projects with EI, SI, PI, II, SPI, SII, OI in agricultural productivity were 72.70%, 43.92%, 55.19%, 15.87%, 32.79%, 11.71%, 7.13%, respectively (Fig 9). The rate of the projects with EI was high in most provinces, specifically, in 25 provinces where more than 50% of the projects were effective on EI of agricultural productivity. The rates of the projects with EI were low in Jilin and Qinghai, they were 25.28% and 35.71%, respectively. The distribution of the effectiveness of FC on PI was similar to that on EI. The effectiveness of FC on SI was prominent in eastern and southeast China. However, the projects located in northern and northwest China (e.g., Jilin, Heilongjiang, Ningxia, Gansu) showed a low effectiveness on SI. In addition, the projects were more effective on SPI in southeast China than the other areas.

The rates of the projects with II, SPI, OI in agricultural productivity were lower than the rates of the projects with other types of agricultural productivity improvement. The effectiveness of FC on the above three types of agricultural productivity improvement was remarkably more prominent in eastern and middle China, such as Tianjin, Shandong, Jiangsu, Shanghai, Zhejiang. Tianjin had the most effective expression in the three types of agricultural productivity improvement, where the rate of the projects with OI was 32.76%. More than two third provinces where the rate of the projects with OI was lower than the mean level in the whole country. The projects in Jilin, Heilongjiang, Ningxia, Qinghai and Hainan were all non-effective on both SII and OI of agricultural productivity.

## Discussion

### Potential factors associated with the effectiveness of farmland consolidation on agricultural productivity improvement

This study present that FC is an effective and feasible means for improving agricultural productivity, which is consistent with findings in previous studies [17, 19, 58]. FC can improve agricultural productivity in a given socioeconomic and technologic status, the optimal improvement in agricultural productivity is obtained under optimum land management practice in a given region, which can be achieved through land consolidation measures [17, 59, 60].

The effectiveness of FC on improving agricultural productivity was more prominent in areas with better socioeconomic conditions (e.g., higher per capita GDP and dense urbanization), such as southeast China and southern China, which are able to provide a favorable capital and technical foundation for FC [61]. FC in these areas was supported by more sufficient investment and advanced land engineering measures, high-quality farmland infrastructures (e.g., irrigation and drainage installations) have been constructed for efficient farming. Moreover, these areas are located in the geographic zone with a large share of plains and suitable climatic conditions (e.g., moderate temperature and precipitation) for supporting the development of agricultural production.

In general, FC has provided good prospects for improving agricultural productivity. However, a relevant share of FC projects have shown to be non-effective on improving agricultural productivity in the northern China. Land quality was initially high and the area of farmland was large in these areas, but they have experienced frequent chilling damage as they are located in cold temperate zone. It is difficult for FC to improve the ability of farmland to resist natural disaster in a short time [60, 62]. Limited by extreme climate and highland terrain as well as the low-level socioeconomic development, the effectiveness of FC in west China also showed a low status on improving agricultural productivity. Moreover, land engineering measures could disturb the stabilization of ecosystems (e.g., soil disturbance, water surface reduction) [63, 64], which leads to constraints for improving agricultural productivity potential in eastern China.

### Implications for improving the effectiveness of farmland consolidation

FC have important impacts on agricultural production, contributing to higher level of agricultural productivity (especially in the lowlands), but also to soil disturbance in the areas, especially may lead to soil erosion in the highlands. Additionally, the improper method of selecting the re-parcelling plots has a negative impact on the effectiveness of FC. This suggests the importance of a demand-driven planning and design for such FC projects based improved approach to ensure that location-specific factors can be adequately considered [65, 66]. All submitted FC projects should be evaluated in terms of desirable outcomes estimated on the basis of location-specific factors. This would probably eliminate areas where FC would not bring a significant improvement on agricultural productivity. Furthermore, the reorganization of a cluttered plot pattern should be carried out in sequence, starting in areas without a costly post-consolidation development [58].

The other reason would be responsible for non-effective FC in China is the funding strategy. In the participation of FC, cost is commonly practiced and is considered to be an essential influencing factor on the effectiveness of FC [67]. Previous studies have proved that the investment was significantly higher in middle and eastern China than in the western and northern China, and the central and local government were the main even the only fund provider [68, 69]. Thus, a multi-financing system with diverse financial sources should be established to improve the effectiveness of FC. Besides state budget, incentive measures also should be adopted to raise social capital from financial institutions (e.g., bank, insurance agency, trust fund) and rural collective economic organization to support the implementation of FC [58, 68].

### Limitations and uncertainties

The four indictors captured agricultural productivity change over multiple years using NDVI time series data, but rest on reliable identification of NDVI data. Although the quality assessment suggests the data are reliable (RI ≤1), uncertainty for some projects (e.g., with small areas) may be higher than for others [26]. However, it should be noted that this study focuses on the direction of agricultural productivity change after FC, rather than estimating crop yield and its change. The difference of NDVI before and after FC was calculated to characterize the effectiveness of agricultural productivity improvement, this relative approach aimed to reduce the uncertainties of NDVI. Moreover, the study was carried out at a national scale, NDVI monitoring error caused by man-made interference (e.g., farmland abandonment, planting structure adjustment) is relatively small or even negligible and have not been taken into account in the study.

## Conclusions

In this study, we used MODIS-NDVI time series data to extract four indicators to characterize agricultural productivity change of 4442 FC projects completed between 2006 and 2010. Limited due to the lag of vegetative cover reestablishment on disturbed soil after FC, it is difficult and inaccurate to use NDVI data to evaluate the effectiveness of the FC projects completed recently. As such, we introduced a machine learning algorithm named SVM to predict the same four characteristics of agricultural productivity change of 3603 projects completed between 2011 and 2013. We found that FC in China showed an overall effective status, 72.70% of all 7505 projects were effective on the elementary productivity improvement. Moreover, 7.13% of the projects were effective on improving all four productivity characteristic indicators. The effectiveness of FC was more prominent in southeast and eastern China. This is mainly attributed to the suitable climate and high-level socioeconomic development for supporting FC in the areas. However, it is also noteworthy that 27.30% of the total projects still were ineffective in increasing the productivity level. This is mainly attributed to ecosystem disturbance produced by FC as well as extreme climatic condition and low-level investment socioeconomic development in the areas. Thus, we propose that location specific factors and diverse funding sources should be considered in FC. The findings in this study provide a reference for policy-makers to arrange FC projects reasonably and formulate land consolidation planning in a proper way in future.

## Supporting information

S1 Fig The locations of the projects completed between 2006 and 2013 in China. (TIF)
