# Supplementary figures and images for: Evaluating and predicting the effectiveness of farmland consolidation on improving agricultural productivity in China
Source: PLoS One. 2018 Jun 6;13(6):e0198171. doi: 10.1371/journal.pone.0198171 (PMC5991407; doi:10.1371/journal.pone.0198171)

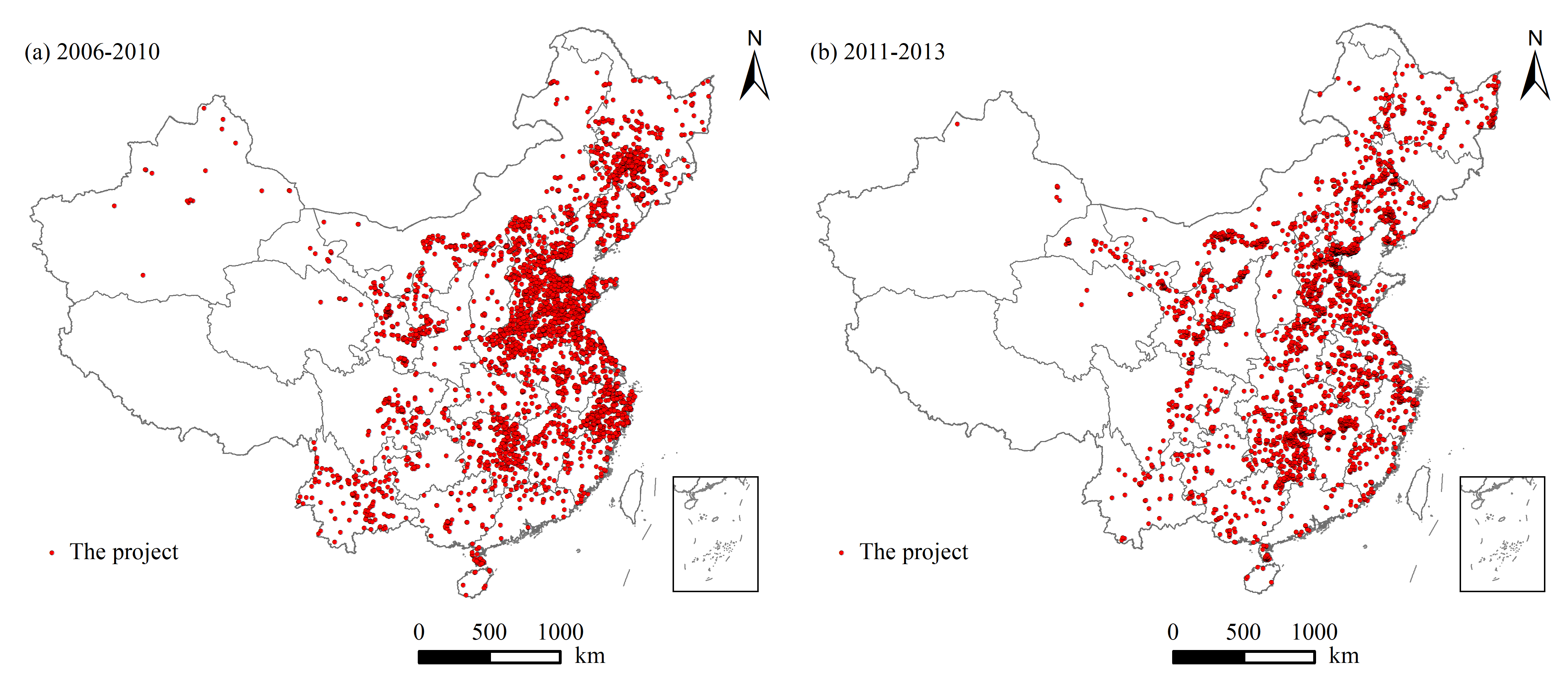

Supplement: S1 Fig — (TIF) [file pone.0198171.s001.tif]
